# Black Gram (*Vigna mungo* L.) Husk as a Source of Bioactive Compounds: An Evaluation of Starch Digestive Enzyme Inhibition Effects

**DOI:** 10.3390/foods14050846

**Published:** 2025-02-28

**Authors:** Chi Vo Ngoc Dinh, Nopparat Prabsangob

**Affiliations:** Department of Product Development, Faculty of Agro-Industry, Kasetsart University, Bangkok 10900, Thailand

**Keywords:** antioxidant activity, α-amylase inhibition, α-glucosidase inhibition, black gram husk, flavonoids, phenolics, saponin

## Abstract

This research recovered bioactive compounds from black gram husk (BGH, a by-product of sprout processing) using different ethanol concentrations and maceration times. Based on the results, the highest phenolic and saponin contents were recovered using an 80% ethanolic solution for 3 h, with the extract having both antioxidant and starch digestive enzyme inhibition effects. The major bioactive compounds present in the extract were gallic acid, gentisic acid, ferulic acid, and vitexin. The extract from BGH had an effective binding affinity to α-glucosidase, resulting in a potent ability of the extract to delay enzyme activity. Based on in vitro starch digestion using cooked rice as a model, adding the extract (10 mg/mL) increased the resistant starch content (from 53.9% to 55.9%) and lowered the estimated glucose index (from 83.1% to 81.0%) as compared to the control without the extract. Based on the overall results, the BGH extract could be promising as a functional ingredient with antioxidant activity and the ability to control postprandial blood glucose levels in the development of healthy food products.

## 1. Introduction

Legumes are one of the important staple foods globally due to their availability and high nutritional value. Black gram (*Vigna mungo* L.), particularly for its sprouts, is popularly consumed in several regions, including Asia and Western countries [1]. The sprouting process allows several biochemical changes, resulting in the improved nutritive quality of plant seeds, including by promoting protein digestibility, increasing the availability of macronutrients, and diminishing antinutrient factors [2]. In the commercial sprouting process, black gram husk (BGH) is discarded as a by-product that has only low-value uses, such as animal feed and filling material for pillows and mattresses. The sprouting process takes a short time of only a few days, so the husk left from the process is always free from chemical treatments. Notably, the seed husks of some legumes are a good source of phytochemicals with bioactive qualities, such as antioxidant, antidiabetic, anti-obesity, antimicrobial, and anticancer activities [3,4]. However, there is only limited information available on the utilization of BGH material left from a sprouting process.

Diabetes mellitus (DM) is a metabolic disorder characterized by a chronic high blood glucose level related to insulin resistance and insufficient insulin production [5]. The number of adults with diabetes has nearly tripled from 151 million in 2000 to 451 million in 2017 and is expected to reach 693 million by 2045 [6]. Type 2 DM—non-insulin-dependent DM—is the most common form, accounting for more than 90% of all diabetic patients [7]. Several synthetic drugs, such as Acarbose, are generally used to inhibit the starch digestive enzymes for controlling postprandial hyperglycemia in patients with Type 2 DM [8]. However, these synthetic compounds are costly to produce and have side effects such as liver disorders, flatulence, and abdominal cramping [8]. Therefore, an alternative option to treat patients suffering from DM may be to use phytochemicals that can delay starch digestive enzyme activity.

The extraction of bioactive compounds from plant materials depends on both intrinsic (e.g, plant types and growing conditions) and extrinsic (e.g, extracting solvents and the used conditions) factors [9,10,11,12,13,14,15]. Several bioactive compounds could be recovered from legumes such as polyphenols from cowpea [9], adzuki bean, mung bean [16], and lentil seed coat [4], as well as saponin from lupin and soybean [17]. To obtain the plant extracts with the highest content of the bioactive compounds, and thereby effective bioactivities, the extraction condition—e.g., material-to-solvent ratio, extraction temperature, and time—has to be optimized. Usually, organic solvents such as methanol are effective extracting media. Nevertheless, organic solvents are always harmful to health and the environment [4]. Recently, there has been increasing interest in the recovery of bioactive compounds from natural resources using green solvents such as water and ethanol, owing to their safety and sustainability [4,17].

The present work aimed to exploit BGH, a by-product from a sprouting process, as a source of food ingredients with bioactivities, especially for its starch digestive enzyme inhibition effect. First, the effective conditions were studied to obtain extracts from the BGH with potent bioactivity. Then, the effects of the extract on the activity of starch digestive enzymes were elucidated before determining the influence of BGH extracts on in vitro starch digestibility. The outputs from this research could provide useful information on practical applications of BGH as a source of bioactive compounds for the development of functional food products.

## 2. Materials and Methods

### 2.1. Materials and Chemicals

BGH was kindly supplied by Talingchan Bean Sprouts (Bangkok, Thailand). Analytical-grade reagents of gallic acid, (+)-catechin, 6-hydroxy-2,5,7,8-tetramethylchroman-2-carboxylic acid (Trolox), caffeic acid, ferulic acid, gentisic acid, protocatechuic acid, syringic acid, 2,2-diphenyl-1-picrylhydrazyl (DPPH), Folin–Ciocalteu phenol reagent, 2,2-azinobis-3-ethylbenzothiazoline-*6*-sulfonic acid (ABTS), 2,4,6-tri(2-pyridyl)-*s*-triazine (TPTZ), 3,5-dinitrosalicylic acid (DNS), vitexin, diosgenin, *p*-nitrophenyl α-D-glucopyranoside (pNPG), and porcine pancreatic α-amylase (α-Amy) (EC 3.2.1.1, type VI) were purchased from Sigma Chemical Co. (St. Louis, MO, USA). Analytical-grade solvents of Acarbose and α-glucosidase (α-Glu) were products from Thermo Scientific Chemicals Co. (Shanghai, China) and Megazyme Ltd. (Bray, Ireland), respectively. HPLC-grade solvents, including glacial acetic acid, acetonitrile, and sulfuric acid, were purchased from QRëc chemical (Auckland, New Zealand). Other chemicals involving starch-soluble NaNO_2_, Na_2_CO_3_, NaOH, vanillin, AlCl_3_, FeCl_3_, NaCl, MgCl_2_, CaCl_2_, K_2_S_2_O_8_, D-glucose, and CH_3_COONa were analytical-grade reagents from KemAus Co. (Cherrybrook, Australia).

### 2.2. Extraction of Bioactive Compounds from BGH

The BGH was washed several times with water to remove foreign matter and then dried at 60 °C until the moisture content was less than 10%. Next, the dried BGH was ground in a blender and passed through a sieve (60 mesh, 250 μm). The BGH powder was stored in a plastic bag at 8 ± 2 °C for less than 3 months before use. The chemical composition of the BGH was determined according to the standard methods [18], and the result involved crude protein (9.27 ± 0.23%), crude fat (0.33 ± 0.07%), moisture (9.54 ± 0.05%), and ash (2.83 ± 0.04%).

**Figure 1:**
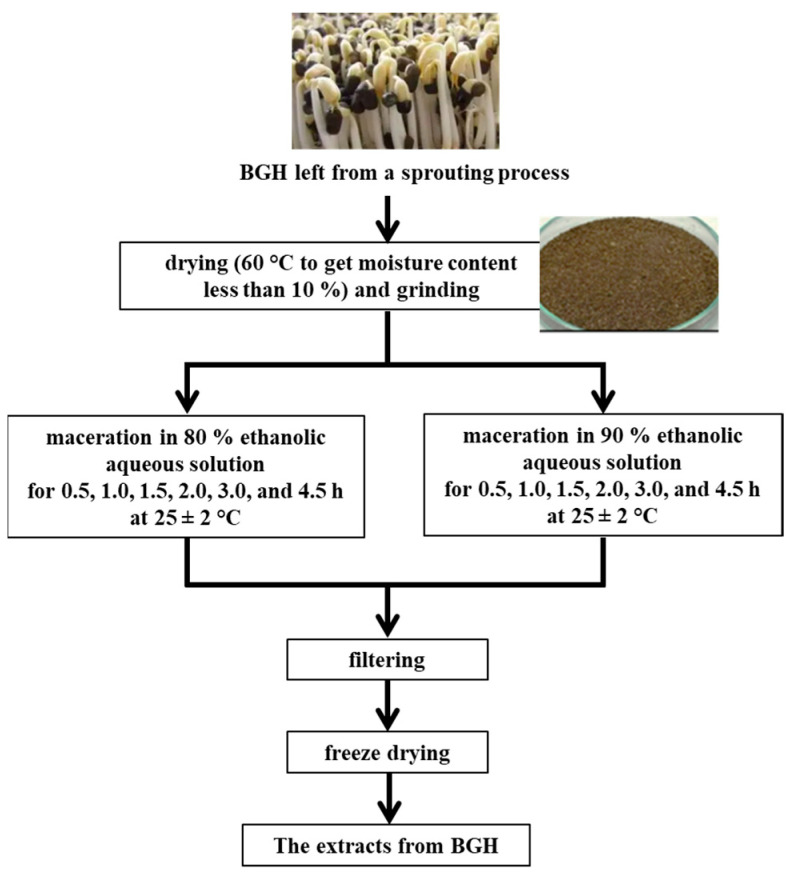
Diagram for the preparation of the extract from BGH.

Extraction of bioactive compounds from the BGH was performed according to the method of Thoo et al. [19] with some modification, and the diagram of the extraction is represented in Figure 1. The BGH powder was added with aqueous ethanolic solution (at a solid-to-liquid ratio of 1:10) at varying concentrations (80% or 90%, *v*/*v*), and macerated for different times (0.5, 1.0, 1.5, 2.0, 3.0, and 4.5 h) at ambient temperature (25 ± 2 °C) with continuous shaking. After reaching the designated time, the supernatant was collected by passing it through a filter paper and evaporating it under a reduced pressure (R-300; Büchi Labortechnik AG; Flawil, Switzerland), and then it was freeze-dried to obtain the extracts for further analysis.

### 2.3. Antioxidant Activity and Starch Digestive Enzyme Inhibition Effect of Extracts from BGH

#### 2.3.1. Total Phenolic Content (TPC)

The TPC was determined based on a Folin–Ciocalteu assay according to the method of Xu and Chang [20], with a slight modification. The methanolic solution of the extract was reacted with Folin–Ciocalteu reagent and Na_2_CO_3_ solution. After incubation for 2 h in the dark, the absorbance at 765 nm was recorded using a UV–visible spectrophotometer (UV 1900; Shimadzu; Kyoto, Japan). The TPC was calculated based on a standard curve of gallic acid and quantified as a gallic acid equivalent (GAE).

#### 2.3.2. Total Flavonoid Content (TFC)

The TFC was quantified as per the method of Xu and Chang [20], with a slight modification. The methanolic solution of the extract was added to the NaNO_2_ solution. After incubating at room temperature, the mixture was reacted with an AlCl_3_·6H_2_O solution, NaOH solution, and deionized water before measuring the absorbance at 510 nm. The TFC was calculated based on a standard curve of (+)-catechin and reported as a catechin equivalent (CE).

#### 2.3.3. Total Saponin Content (TSC)

The TSC was measured using the vanillin–sulfuric acid method as described by Singh et al. [21]. Briefly, the methanolic solution of the extracts was reacted with a vanillin ethanolic solution and sulfuric acid. After mixing well, the mixture was heated (60 °C, 10 min) and then immediately cooled in an ice bath before measuring the absorbance at 544 nm. The TSC was quantified based on a standard curve of diosgenin and reported as a diosgenin equivalent (DE).

#### 2.3.4. DPPH Radical Scavenging Activity

The DPPH radical scavenging capacity of the extracts was evaluated according to Xu and Chang [20], with a slight modification. The methanolic solution of the extract was added to the DPPH solution at a ratio of 1:19, *v*/*v*. The extract solution (2 mg/mL) was mixed vigorously and left at ambient temperature in the dark for 30 min. Then, the absorbance of the mixture was recorded at 517 nm. The DPPH radical scavenging ability was quantified based on a standard curve of Trolox and reported as a Trolox equivalent (TE).

#### 2.3.5. ABTS Radical Scavenging Activity

The ABTS radical scavenging activity of the extracts was determined according to the method of Singh et al. [22], with a slight modification. First, the ABTS radical solution was generated by mixing ABTS with the K_2_S_2_O_8_ solution and incubating it at ambient temperature for 12–16 h in the dark. The ABTS radical solution was diluted with ethanol to obtain an appropriate absorbance at 734 nm before reacting it with the methanolic extract solution (2 mg/mL) at a ratio of 1:10, *v*/*v*. The absorbance at 734 nm was recorded, and ABTS radical scavenging activity was quantified based on a standard curve of Trolox and reported as TE.

#### 2.3.6. Ferric Reducing Antioxidant Power (FRAP) Activity

The FRAP of the extracts was determined according to Xu and Chang [20], with a slight modification. The FRAP working reagent was prepared by mixing acetate buffer, FeCl_3_·6H_2_O solution, and TPTZ before incubation at 37 °C for 10 min. The methanolic solution of the extracts (2 mg/mL) was reacted with the FRAP working solution at a ratio of 1:30, *v/v*, and left at ambient temperature in the dark for 30 min before measuring the absorbance at 593 nm. FRAP was quantified based on a standard curve of Trolox and reported as TE.

#### 2.3.7. α-Amy Inhibition Activity

The ability of the extracts to inhibit α-Amy activity was estimated according to Dong et al. [23], with a slight modification. The extract solution (2 mg/mL) was prepared using a phosphate buffer (10 mM, pH 6.9) with the presence of NaCl (6 mM) and α-Amy (1.0 U/mL, 200 µL). After incubating at 25 °C for 10 min, a starch solution (0.25%, 0.4 mL) was added. The reaction was carried out at 37 °C for 5 min and terminated by adding DNS reagent (1%, 1.0 mL). After incubating in boiling water for 5 min, the mixture was immediately cooled to room temperature and deionized water (10 mL) was added before measuring the absorbance at 540 nm (A_sample_). The control was prepared in the same manner using the buffer solution instead of the extract. Mixtures without added enzyme, sample extract, and Acarbose were used as blanks. The enzyme inhibitory effect was calculated using Equation (1):

(1) $$Enzyme\;inhibition\;effect\;(\%)=\frac{\left[A_{control}-\left(A_{sample}-A_{blank}\right)\right]\times 100}{A_{control}}$$

#### 2.3.8. α-Glu Inhibition Capacity

The ability of the extracts to delay α-Glu activity was estimated according to Dong et al. [23], with a slight modification. Briefly, the extract solution (2 mg/mL) was prepared using a phosphate buffer (10 mM, pH 6.9) and added to an α-Glu solution (0.2 U/mL, 0.5 mL) before incubation at 37 °C for 10 min. Then, the sample was reacted with a pNPG solution (1 mM, 0.5 mL) and incubated at 37 °C for 20 min before adding Na_2_CO_3_ (0.2 M, 1.6 mL). Next, the absorbance was read at 405 nm (A_sample_). For the control, the buffer was used instead of the extract. Mixtures without added enzymes, the extract form of BGH, and Acarbose were used as blanks. The enzyme inhibitory effect was calculated using Equation (1).

The extraction conditions providing the extract with the most potent antioxidant and starch digestive enzyme inhibition effects were selected to prepare the BGH bioactive extract (BGH-BE).

### 2.4. Bioactive Compounds of BGH-BE

The major compounds available in the BGH-BE were determined according to Mu et al. [24] using high-performance liquid chromatography (HPLC; Agilent 1100 series; Palo Alto, CA, USA) equipped with a C18 column (250 mm × 4.6 mm, 5.0 μm). Mobile phase A (2% glacial acetic acid) and mobile phase B (acetonitrile) were used with gradient conditions of 0–5 min, 3% B; 5–15 min, 3–10% B; 15–25 min, 10–25% B; 25–35 min, 25–30% B; 35–40 min, 30–3% B; and 40–42 min, 3% B at a flow rate of 1 mL/min, and a temperature of 30 °C. The UV-Vis detector at wavelengths of 280 nm for phenolic compounds and 337 nm for flavonoid compounds was employed. Selected standards were used: caffeic acid, chlorogenic acid, cinnamic acid, ferulic acid, gallic acid, gentisic acid, protocatechuic acid, syringic acid, and vitexin.

### 2.5. Effect of BGH-BE on Activity of Starch Digestive Enzymes

The effects of BGH-BE on the activity of the starch digestive enzymes were elucidated based on measurements.

#### 2.5.1. Kinetic Inhibition on Enzyme Activity

The kinetic inhibition of BGH-BE on α-Amy activity was examined according to the method described above in Section 2.3.7 by introducing the BGH-BE (0–1.6 mg/mL) to substrates of different starch solutions (0–2%, *w*/*v*). The α-Glu activity was determined by reacting the BGH-BE (0–1.6 mg/mL) with the substrate of pNPG solutions (0–1.2 mg/mL) according to the method described above in Section 2.3.8. The kinetic inhibition mechanisms of the extract on enzyme activity were determined based on the Lineweaver–Burk equation, as shown in Equation (2) [23]:

(2) $$\frac{1}{V}=\frac{1}{V_{max}}+\frac{K_{m}}{V_{max}}\times \frac{1}{S}$$

where V and V_max_ are the initial rate and the maximum rate of the reactions, respectively; K_m_ is the Michaelis–Menten constant; and S is the substrate concentration.

#### 2.5.2. UV Absorption Spectroscopy

The effects of the BGH-BE (0.1–1 mg/mL, 0.25 mL) on the UV-Vis spectra of α-Amy (1.0 U/mL, 3 mL) and α-Glu (0.2 U/mL, 3 mL) were investigated in the absorbance range 190–400 nm [25].

#### 2.5.3. Fluorescence Quenching

The BGH-BE solutions at varying concentrations (0.1–1 mg/mL, 0.25 mL) were added with α-Amy (1.0 U/mL, 3 mL) or α-Glu (0.2 U/mL, 3 mL) for 3 min before recording the fluorescence spectra at the emission wavelengths (λ_em_) of 300–500 nm and excitation wavelength (λ_ex_) of 280 nm using a spectrofluorometer (FluoroMax Plus; Horiba; Japan). Then, the fluorescence quenching of the extract was analyzed using the Stern–Volmer equations, as shown in Equations (3) and (4) [25]:

(3) $$\frac{F_{0}}{F}=1+K_{q}\tau_{0}\left[Q\right]=1+K_{sv}[Q]$$

(4) $$log\frac{F_{0}-F}{F}=logK_{a}+n\;\log[Q]$$

where F_0_ and F are the fluorescence intensities with the presence and absence of the extract, respectively, K_q_ and K_sv_ are the biomolecular quenching constants and Stern–Volmer quenching constants, respectively, $\tau_{0}$ is the lifetime of fluorophore, K_a_ is the binding constant, n is the number of binding sites of the enzymes, and Q is the extract concentration.

### 2.6. Effect of BGH-BE on In Vitro Starch Digestion

The effect of the BGH-BE on in vitro starch digestion was observed using cooked rice as a food model. The grains of rice (*Oryza sativa* L.) were soaked in distilled water with a solid-to-liquid ratio of 1:1.5 before cooking using a rice cooker (KSH-D06; Sharp; Sakai, Japan) with a program consisting of 16 min heating, 10 min ripening, and 30 min of cooling to room temperature. Then, the cooked rice was analyzed.

#### 2.6.1. Total Starch Content (C_TS_)

The C_TS_ was determined using the DNS method [26]. The freeze-dried cooked rice powder (100 mg) was treated with sulfuric acid (1.5 N, 10 mL) in a boiling water bath for 3 h before cooling and neutralizing using NaOH (0.25 M). The C_TS_ was determined using a DNS method with D-glucose as a standard and then quantified using Equation (5):

(5) $$C_{TS}\;\left(\%\right)=\frac{C_{TG}\times 0.9}{W_{T}}\times 100$$

where C_TG_ is the percentage of total glucose in the hydrolysate and W_T_ is the sample weight.

#### 2.6.2. In Vitro Starch Digestion

The cooked rice was homogenized to obtain a slurry, before introducing in vitro digestion according to Englyst et al. [27]. Briefly, the slurry of cooked rice (1.0 g) was suspended in acetate buffer (0.2 M, pH 6.0, 9 mL) containing CaCl_2_ (200 mM) and MgCl_2_ (0.5 mM). The BGH-BE at varying contents (2, 5, or 10 mg/g) was added to the mixture before incubating at 37 °C for 10 min. Then, the mixed enzyme solution (10 U/mL of α-Amy and 18 U/mL of α-Glu) was introduced for digestion at 37 °C for 3 h. During the digestion, a small aliquot of the hydrolysate was periodically withdrawn and added with ethanol to inactivate the enzymes. The unreacted starch residue was separated using centrifugation and the glucose content was quantified using the DNS method. The contents of the different starch fractions involving rapidly digestible starch (RDS; the hydrolyzed portion within 0–20 min), slowly digestible starch (SDS; the hydrolyzed portion within 20–120 min), and resistant starch (RS; the undigested portion after 120 min) were calculated based on Equations (6)–(8), respectively:

(6) $$RDS\left(\%\right)=\frac{({Glu}_{20}-{Glu}_{0})\times 0.9}{W_{s}}\times 100$$

(7) $$SDS\left(\%\right)=\frac{({Glu}_{120}-{Glu}_{20})\times 0.9}{W_{s}}\times 100$$

(8) $$RS\left(\%\right)=\frac{[W_{s}-\left(RDS+SDS\right)]\times 0.9}{W_{s}}\times 100$$

where Glu_0_, Glu_20_, and Glu_120_ are the amounts of glucose released within 0, 20, and 120 min of the digestion, respectively, and Ws is the weight of the starch sample.

The percentage of starch hydrolysis was estimated using Equation (9), and a first-order equation model was applied to elucidate the kinetics of starch hydrolysis using Equation (10) [28]:

(9) $$Starch\;hydrolysis\;\left(\%\right)=\frac{G_{t}\times 0.9}{W_{s}}\times 100$$

C_t_ = C_∞_ (1 − e^−kt^) (10)

where G_t_ is the released glucose content at digestion time t, k is the kinetic constant, C_t_ is the proportion of hydrolyzed starch at time t, and C_∞_ is the equilibrium starch concentration.

From the in vitro digestion, the hydrolysis index (HI) was calculated based on the estimated k and C_∞_ values. The HI was calculated using the area under the digestibility curve (AUC) between the initial (t_0_) and selected (t_x_) time points, with the AUC obtained from the first-order equation bounds between the times t_0_ and t_x_ as per Equation (11). HI was calculated based on the AUC ratio of the sample and a reference product (white bread) using Equation (12). The ingested carbohydrates based on a postprandial level of blood glucose were determined using the estimated glycemic index (eGI) according to Equation (13) [29]:

(11) $$AUC=C_{\infty }\left(t_{x}-t_{0}\right)+\frac{C_{\infty }}{k}[\left(e^{-kt_{x}}-e^{-kt_{0}}\right)]$$

(12) $$HI\;(\%)=\frac{{AUC}_{sample}}{A{UC}_{white\;bread}}$$

eGI = 39.71 + 0.549 HI(13)

### 2.7. Statistical Analysis

All determinations were conducted in triplicate, and the data were presented as mean ± standard deviation values. Analysis of variance was performed using Duncan’s test, and statistical significance was tested at the 95% confidence level. Data analysis was carried out using the SPSS software package (SPSS Statistics, version 22.0; IBM Corporation; Armonk, NY, USA).

## 3. Results and Discussion

### 3.1. Extraction of Bioactive Compounds from BGH

**Figure 2:**
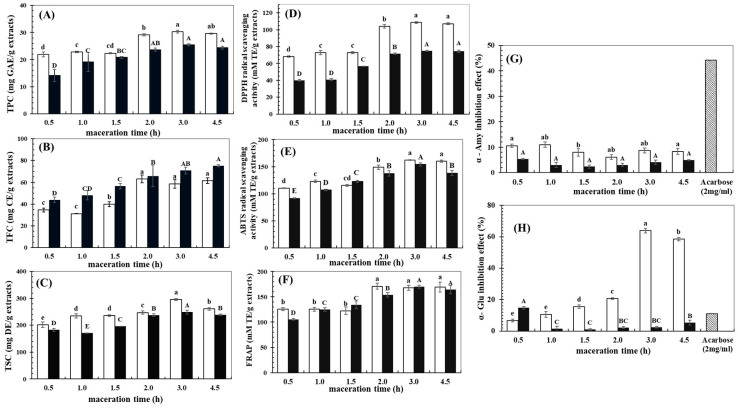
Effects of maceration time on (**A**) TPC, (**B**) TFC, (**C**) TSC, (**D**) DPPH radical scavenging ability, (**E**) ABTS radical scavenging ability, (**F**) FRAP, (**G**) α-Amy inhibition effect, and (**H**) α-Glu inhibition effect of extracts prepared from BGH using 80% (open bars) and 90% (closed bars) ethanolic solutions. In each subfigure, different lowercase and capital letters indicate significant differences between means for the extracts using 80% and 90% ethanolic solutions, respectively (*p* ≤ 0.05). The concentration of the ethanolic solutions significantly affected all measured parameters (*p* ≤ 0.05). In Figure 2G,H, Acarbose at a corresponding concentration with extracts was used as a reference for comparison.

Ethanol concentration and maceration time significantly affected the contents of bioactive compounds recovered from BGH (Figure 2). Generally, the TPC, TFC, and TSC values of the extracts increased with prolonged maceration time before reaching a plateau at around 2–3 h after extraction. Increasing the maceration time promoted contact between the solute and solvent and enhanced diffusion of the solute from the plant cells to the solvent, thus encouraging a release of bioactive compounds [30]. However, with excessive maceration time, equilibrium diffusion between the solute in the plant matrix and the bulk solution might be reached [10], resulting in no further improvement effect on the recovered content of the bioactive compounds. Similar behavior was also reported for the bioactive compound extraction from other plant sources such as starfruit residues [11] and peach [10].

The 80% ethanolic solution provided the extracts with higher TPC and TSC values, whereas the TFC was greater from extracts recovered using 90% ethanolic solution. These dissimilar ethanol concentrations affected the solvent’s physical properties (polarity, density, dynamic viscosity, and dielectric constant), thereby differentiating the recovered compounds [12]. The polarity indices of ethanol and water are 5.2 and 7.0, respectively [13]. Therefore, lowering the ethanol content led to a higher polarity of the extracting solvent. Phenolic compounds always consist of a carboxylic group joined to a benzene ring [12]. Consequently, extraction using the lower ethanol content of 80% might produce more extractable polar phenolic compounds compared to the higher ethanol content. These results were in agreement with other studies on recovering phenolic compounds from several plants such as *Averrhoa bilimbi* [13], *Toona senesis* [14], olive leaves [15], and legumes [20]. Saponins are the hydro-soluble compounds consisting of an aglycone structure attached to a saccharide [17], and in most plants, saponins are present as polycyclic aglycones covalently bonded with one or more sugar moieties called sapogenin [31]. It has been suggested that ethanolic solutions with concentrations of 50–80% could be effective at recovering saponins from several plant materials [32]. On the other hand, flavonoids are bioactive compounds with a basic chemical structure of a C6-C3-C6 phenyl benzopyran backbone substituted with varying residues, resulting in their diverse polarity [33]. In the present study, the higher TFC value of the extracts prepared using the 90% ethanolic solution might have been due to the polarity of the solvent being appropriate to dissolve the flavonoids present in the BGH. Increasing the concentration of the ethanolic solution from 70 to 96% led to the improved flavonoid recovery efficacy of *Toona sinensis* leaves [14] and other legumes [20].

Antioxidant activity of the extracts was evaluated through both primary (i.e., DPPH and ABTS radical scavenging abilities) and secondary (i.e., FRAP) mechanisms. DPPH radicals, dissolving in organic solvents and providing the absorption at 517 nm, are appropriate for assessing the hydrophobic antioxidant system, whereas the blue-green ABTS^+^ radicals with the absorption peak at 734 nm are suitable for evaluating antioxidant activity in both hydrophilic and lipophilic environments [34]. The FRAP assay relates to the capability of the bioactive compounds to reduce the Fe^3^^+^-TPTZ complex to its ferrous form as Fe^2^^+^-TPTZ under acidic conditions [20]. In this study, increasing the maceration time generally improved the antioxidant activities of the extracts through both primary DPPH (Figure 2D) and ABTS (Figure 2E) free radical scavenging effects, as well as via secondary FRAP (Figure 2F) mechanisms. Generally, extraction using the 80% ethanolic solution produced extracts with greater antioxidant activities than the 90% ethanolic solution.

The appropriate extraction conditions were further investigated by considering the relationship between the content of the bioactive compounds and the bioactivity of the extracts from the BGH using Pearson’s correlation coefficient (r) values, as shown in Table 1. There was a positive correlation between antioxidant activities and the TPC, TFC, and TSC values of the bioactive compounds.

The influences of the extracts from the BGH on the activities of α-Amy (Figure 2G) and α-Glu (Figure 2H) were further evaluated based on a comparison with a standard of Acarbose. The pancreatic α-Amy and small intestinal α-Glu are key enzymes for the digestion of dietary carbohydrate in humans [35]. Therefore, delaying these enzyme activities could lower carbohydrate digestion and suppress glucose absorption and any postprandial hyperglycemia effect [36]. Based on the present study, the extracts showed potent activity to delay α-Glu activity, particularly for the extracts prepared using 80% ethanolic solution for 3 h, which was consistent with their greater TPC and TSC values, as well as the antioxidant activities of the extracts. The present results suggested the importance of phenolic compounds and saponins in inhibiting the activity of α-Glu. In contrast, the extracts produced a rather low delaying effect of α-Amy activity. These contradictory outcomes for the different extracts from the BGH on activities of the studied enzymes might have been due to the dissimilar modes of action of the extracts when interacting with the enzymes [35]. The inhibition mechanism of the extracts from the BGH on α-Amy and α-Glu activities should be studied in more detail.

From Table 1, the bioactive compounds present in the extracts from the BGH were highly and positively correlated with the antioxidant activities and α-Glu inhibition effect, as indicated by the Peason’s correlation coefficient (r) values being greater than 0.7 [37]. Extraction using the 80% ethanolic solution generally produced extracts with higher r values than their counterparts recovered using 90% ethanolic solution, suggesting more efficient bioactivities with the former extracting solution. Considering the extracts prepared using 80% ethanolic solution, the highest correlation of antioxidant and α-Glu inhibition effects was observed for the TPC followed by the TSC, implying a crucial bioactive role of these compounds. The availability of several phenolic compounds in some legumes has been reported for ferulic acid, gentisic acid, gallic acid, and protocatechuic acid [38,39]. These phenolic compounds could produce effective antioxidant ability, particularly a free radical scavenging effect [33,39]. The effective ability of saponins to delay α-Glu activity has been reported in both in vitro and in vivo models [40], with this effect being explained by the binding affinity of saponins to the active sites of α-Glu [41].

Based on the present results, the effective bioactivity of the extract prepared using an 80% ethanolic solution might be attributed to the appropriate polarity of the solvent to recover a cocktail of bioactive compounds from the BGH. Consequently, maceration using an 80% ethanolic solution for 3 h was performed to prepare the BGH bioactive compound extract (BGH-BE) for further study.

### 3.2. HPLC Profiles of Compounds Present in BGH-BE

**Figure 3:**
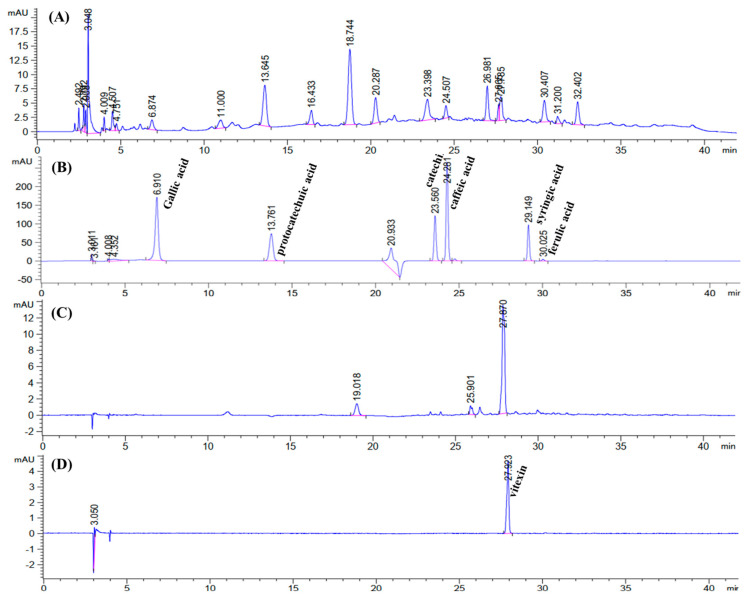
HPLC chromatograms for (**A**) phenolic compounds present in the BGH-BE, (**B**) selected standard for phenolic compounds, (**C**) flavonoids present in the BGH-BE, and (**D**) selected standard for flavonoids. The pink and blue lines represent the base line and signal line, respectively.

The results of the main bioactive compounds identified in the BGH-BE are illustrated in Figure 3. The distinctive phenolic compounds in the BGH-BE, with varying contents, consisted of gentisic acid (ca. 3.87 ± 0.20 mg/g extract), ferulic acid (ca. 2.74 ± 0.07 mg/g extract), catechin (ca. 2.14 ± 0.25 mg/g extract), vitexin (ca. 1.86 ± 0.02 mg/g extract), protocatechuic acid (ca. 1.78 ± 0.08 mg/g extract), and gallic acid (ca. 1.08 ± 0.09 mg/g extract). These results were in agreement with other studies that reported the presence of ferulic acid, gentisic acid, gallic acid, protocatechuic acid, syringic acid, gentisic acid, and vitexin in extracts from BGH derived from a milling process [3] and mung bean seed coats [38,40].

### 3.3. Effects of BGH-BE on Starch Digestive Enzyme Activities

#### 3.3.1. Inhibition Kinetics of α-Amy and α-Glu

**Figure 4:**
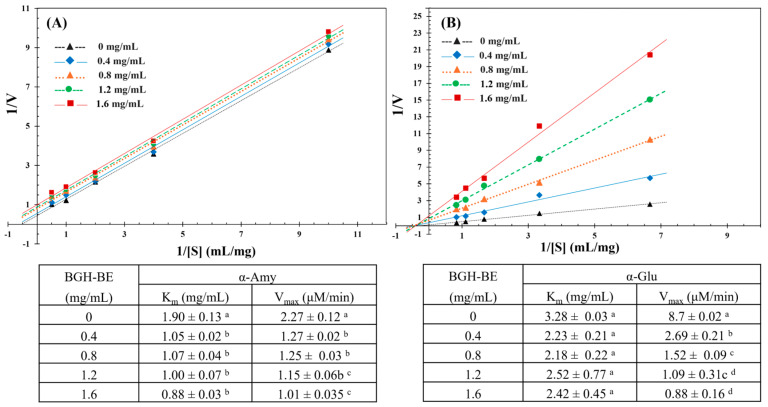
Lineweaver–Burk relationship between BGH-BE at varying concentrations and (**A**) α-Amy and (**B**) α-Glu activities. The inset tables show the Michaelis constant (K_m_) and maximum reaction velocity (V_max_) of the reactions between BGH-BE and the selected enzymes. Different superscripts indicate significant differences in the same column (*p* ≤ 0.05).

The inhibition kinetic mechanism of the BGH-BE on the activities of α-Amy and α-Glu was investigated using Lineweaver–Burk correlations of the reaction rate between the substrates and BGH-BE, as shown in Figure 4. There were clearly dissimilar responses with α-Amy and α-Glu after adding BGH-BE. With α-Amy, K_m_ and V_max_ both decreased with increased BGH-BE concentrations, with the Lineweaver–Burk plot showing a parallel trend (no intersection). This behavior suggested an uncompetitive inhibition mechanism of BGH-BE against α-Amy, in which the extract tended to prefer binding to the enzyme–substrate complex [42,43]. This scenario was correlated with the bioactive compounds extracted from Pinto beans [43]. In contrast with α-Glu, the availability of BGH-BE resulted in trend lines intercepting in the second quadrant and decreased V_m_ and comparatively unchanged K_m_ values with increased BGH-BE concentrations. This trend suggested a mixed-type inhibition effect of the BGH-BE on α-Glu activity [42,44], which included competitively forming an enzyme–inhibitor complex and noncompetitively forming an enzyme–substrate–inhibitor complex to interrupt the enzyme–substrate intermediate [45]. Furthermore, the curves passing through the origin implied a reversible inhibitory effect of the extract [44,46]. Notably, bioactive compounds with a reversible inhibitory mode of action tend to have no in vivo accumulated effect, implying the safety of the compounds for human health [46,47]. The dissimilar inhibition modes of action of the BGH-BE against α-Amy and α-Glu in the present study might have been due to the structural complexity of the bioactive compounds available in the BGH-BE that had differentiated binding manners with the enzymes [45,48].

#### 3.3.2. UV Absorption Spectroscopy and Fluorescence Quenching Assay

**Figure 5:**
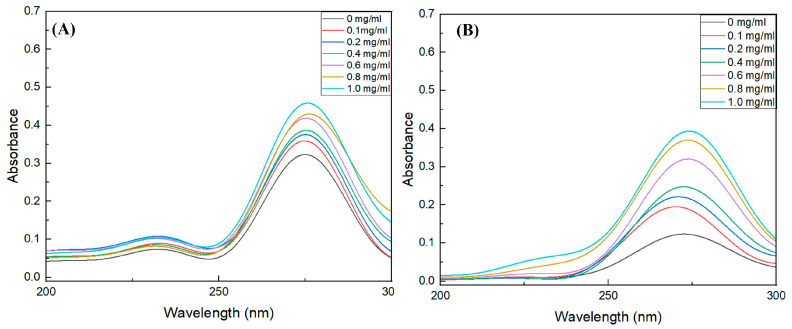
UV spectra of (**A**) α-Amy and (**B**) α-Glu in the presence of the BGH-BE at varying concentrations.

The structural changes in the starch digestive enzymes as affected by adding the BGH-BE were investigated based on the UV spectra of the enzymes (Figure 5). The aromatic amino acids situated at the active site of the enzymes—tryptophan (Trp), tyrosine (Tyr), and phenylalanine (Phy)—were attributed to the UV absorption spectra at the wavelengths in the range of 230–300 nm [48,49]. Alterations in the intensity and shifts in the wavelength of the UV spectra were related to alteration in the microenvironment of the enzymes and could be used to indicate the interaction between the enzymes and the added compounds [48,49]. The present results showed UV spectra with a predominant peak at ca. 250–300 nm for both α-Amy and α-Glu. Adding BGH-BE, particularly at increased levels, led to increased peak intensity, suggesting that the BGH-BE enhanced hydrophobic exposure of the enzymes [48,50]. Furthermore, the spectra of α-Glu had a peak that tended to a slightly blue shift when the BGH-BE was added, suggesting an alteration in the hydrophobicity surrounding the Trp residue [49].

**Figure 6:**
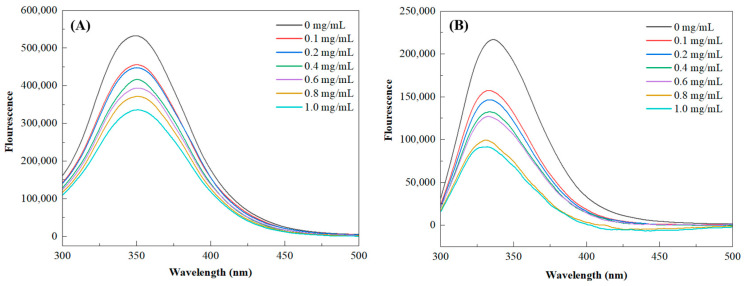
Fluorescence spectra of (**A**) α-Amy and (**B**) α-Glu in the presence of BGH-BE at varying concentrations.

The effects of adding the BGH-BE were investigated based on the fluorescence spectra of the enzymes (Figure 6). The intrinsic fluorescence emission spectra at 340 nm, belonging to Trp and Tyr residues anchoring at the active sites of the enzymes, were predominantly observed for both α-Amy and α-Glu [35,44]. With the presence of BGH-BE, especially at an increased concentration, the peak intensity at 340 nm decreased, suggesting that the enzymes were quenched by the extracts [42,48]. In addition, the fluorescence spectra at 340 nm tended to shift toward a higher wavelength, implying a partial unfolding of the enzymes as affected by adding the BGH-BE, particularly at the increased concentration [42,48]. The binding of the BGH-BE to the enzymes might have occurred through several processes, involving hydrophobic interactions, hydrogen, van der Waals forces, and π-π intermolecular interactions [51]. Polar bonding of phenolic compounds with the functional groups of the proteins could also induce conformational changes in the enzymes [52]. Similar fluorescence quenching behavior to that shown by α-Amy and α-Glu have been reported with some plants such as *Artemisia selengensis* Turcz root [44], *Camellia nitidissima* Chi flower [35], *Lonicera caerulea* berry [46], and coffee [48].

**Figure 7:**
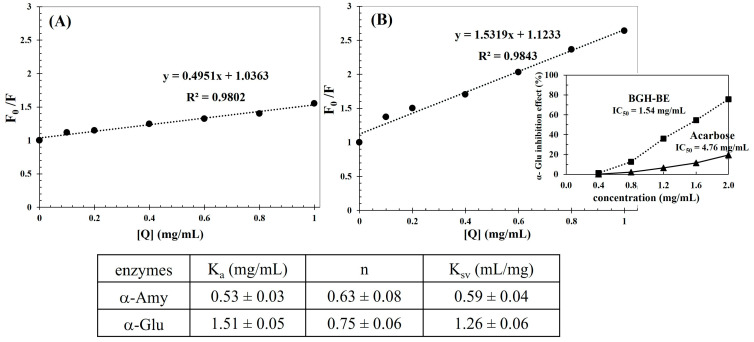
Stern–Volmer relationship between BGH-BE and (**A**) α-Amy and (**B**) α-Glu. The inset table shows the binding constant (K_a_), the number of binding sites per enzyme (n), and the quenching constants (K_sv_) of interaction between BGHE and enzymes. The inset figure of Figure 7B shows BGH-BE concentration dependence on the α-Glu inhibition effect.

The delaying effect of the BGH-BE on activities of the starch digestive enzymes was further studied based on the Stern–Volmer relationships between the fluorescence spectra of the enzymes in the presence of BGH-BE (Figure 7).

From Figure 7, linear relationships with a high coefficient of determination (R^2^ ≈ 0.98) were observed for both enzymes, suggesting a single inhibition site or a single class of inhibition sites for the BGH-BE regarding the enzymes [53]. Higher K_a_, n, and K_SV_ values were clearly evident for the interaction between the BGH-BE and α-Glu, suggesting a greater binding affinity and a stronger interaction of the extract to α-Glu than to α-Amy [54]. This result might explain the greater inhibitory effect of the extracts against α-Glu compared to α-Amy, as shown in Figure 2. The higher affinity of the BGH-BE to α-Glu might have been related to the chemical structures of the bioactive compounds predominantly present in the BGH-BE involving protocatechuic acid, gentisic acid, caffeic acid, catechin, and vitexin. The extracts from the leaves and acorns of *Quercus suber* mainly consist of gentistic acid that produced a potent α-Glu delaying effect, whereas relevant activity against α-Amy was not observed [55]. Compared to Acarbose, catechin showed greater binding affinity to the active site of α-Glu than to α-Amy, with hydrogen bonds and hydrophobic bonding being important interactions between the catechin and the enzymes [56]. Vitexin, a flavonoid with multiple hydroxyl groups, could also interact with α-Glu effectively via hydrogen bond formation [57]. Notably, in the present study, the BGH-BE (IC_50_ of 1.54 mg/mL) had a superior α-Glu delaying effect than the Acarbose (IC_50_ of 4.76 mg/mL) as suggested by the lower IC_50_ value of the extract. The effective inhibition effect of the BGH-BE on α-Glu activity implied a health-promoting benefit of the extract to delay hyperglycemia by lowering the postprandial glucose level [8].

### 3.4. Effect of BGH-BE on In Vitro Starch Digestibility

**Figure 8:**
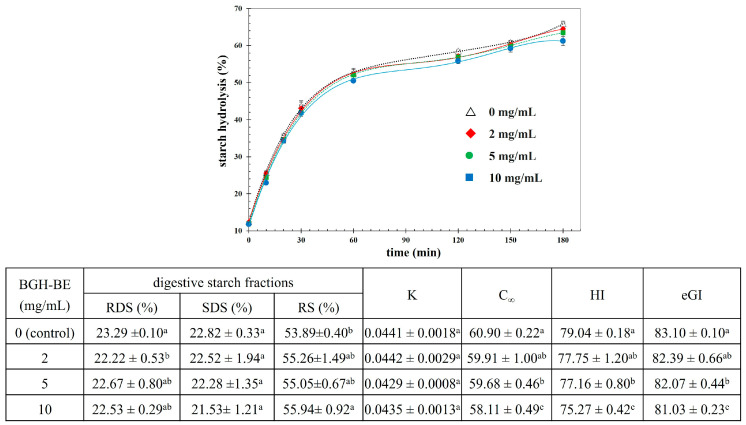
Effect of the BGH-BE at different concentrations on in vitro hydrolysis of cooked rice. The inset table shows the contents of different digestive starch fractions, digestion kinetic constants (Ks), equilibrium starch concentrations (C_∞_), hydrolysis indices (HIs), and estimated glycemic indices (eGIs) of in vitro digested cooked rice as affected by adding BGH-BE. Different lowercase letters indicate significant differences between means in a same column (*p* ≤ 0.05).

The effect of BGH-BE on the in vitro starch digestion was further elucidated using cooked rice as a food model, owing to the importance of rice as a staple food globally. Figure 8 depicts the digestion time dependence based on the degree of starch hydrolysis of the cooked rice added with the BGH-BE at varying concentrations. Generally, the hydrolysis rate increased sharply during the first period of 60 min, before a further gradual increase up to 180 min. Adding BGH-BE, particularly at a sufficient concentration, lowered the starch hydrolysis rate. This could have been due to the extract delaying starch digestive enzyme activities, particularly α-Glu, as suggested by the previous results. Considering the profiles of the digested starch fractions, the BGH-BE had no significant effect on the RDS and SDS contents, whereas the RS content tended to increase with added BGH-BE. RS is the starch fraction that cannot be digested in the small intestine; consequently, RS tends to pass through the large intestine, resulting in its ability to lower the blood sugar level and act as dietary fiber [58,59]. Therefore, based on the present results, the BGH-BE could be beneficial by lowering blood sugar levels and enhancing gut health.

Considering the in vitro starch digestion kinetics, the K value was independent of the BGH-BE concentration, suggesting that the digestion could be defined using a first-order kinetic reaction. Introducing the BGH-BE at a sufficient concentration (≥5 mg/mL) could significantly lower the C_∞_, HI, and eGI values of the digested cooked rice compared to the control without BGH-BE added (see the inset table of Figure 8). These results might be expected due to the binding ability of the BGH-BE to the starch digestive enzymes, as previously observed. In addition, the binding of the BGH-BE to the substrate might also lead to a decreased digestion rate of the cooked rice. Phenolic compounds have a binding affinity with starch mainly via hydrogen bonding [35], which can alter the starch conformation by increasing the ordered crystalline structure, thereby limiting starch digestibility [60]. Furthermore, the phenolic compounds could interact with the hydrophobic helical regions of the starch, resulting in diminished access by the starch digestive enzymes to their substrate [61]. The reported complexation between gallic acid and rice starch [62] and between quercetin and wheat flour [63] could lower the glycemic potential of the model foods.

## 4. Conclusions

BGH, the by-product from a sprouting process, could be used as the source of bioactive compounds. Preparation using an 80% ethanolic solution and a maceration time of 3 h resulted in BGH-BE with effective antioxidant activities and starch digestive enzyme inhibition effects. The predominant bioactive compounds present in the BGH-BE were gentisic acid, ferulic acid, catechin, vitexin, protocatechuic acid, and gallic acid. BGH-BE could bind to α-Glu better than to α-Amy as evident by the UV and fluorescence spectra, resulting in a greater delaying effect of the BGH-BE for α-Glu than for α-Amy. Adding the BGH-BE at a sufficient concentration could significantly increase the nondigested starch fraction and lower the predicted glycemic index of the model food studied through the in vitro model. Therefore, BGH-BE might be applicable as a functional ingredient in the development of food products with health-promoting effects against a metabolic syndrome associated with starch and lipid metabolism.

## Figures and Tables

**Table 1:** Peason’s correlation coefficients (r) for content of bioactive compounds and bioactivities of extracts prepared from BGH using different ethanolic concentrations.

Bioactive Compound		DPPH Radical Scavenging Activity	ABTS Radical Scavenging Activity	FRAP	α-Amy Inhibition Effect	α-Glu Inhibition Effect
80% ethanolic solution	TPC	0.991 **	0.979 **	0.960 **	−0.397	0.837 **
	TFC	0.944 **	0.892 **	0.924 **	−0.422	0.737 **
	TSC	0.950 **	0.914 **	0.919 **	−0.473 *	0.827 **
90% ethanolic solution	TPC	0.867 **	0.909 **	0.906 **	−0.227	−0.647 **
	TFC	0.920 **	0.854 **	0.883 **	0.156	−0.379
	TSC	0.971 **	0.936 **	0.935 **	0.125	−0.328

In the same column, ** indicates a significant difference at *p* ≤ 0.01 and * indicates a significant difference at *p* ≤ 0.05.

## Data Availability

The original contributions presented in this study are included in the article. Further inquiries can be directed to the corresponding author.
